# An Ultrasensitive Picric Acid Sensor Based on a Robust 3D Hydrogen-Bonded Organic Framework

**DOI:** 10.3390/bios12090682

**Published:** 2022-08-25

**Authors:** Weiwei Jiang, Lingling Xia, Dan Li, Pengyan Wu, Tongtong Zou, Xingcheng Yuan, Wen Wei, Jian Wang

**Affiliations:** Jiangsu Key Laboratory of Green Synthetic Chemistry for Functional Materials, School of Chemistry and Materials Science, Jiangsu Normal University, Xuzhou 221116, China

**Keywords:** hydrogen-bonded organic framework, luminescent sensor, picric acid, ultrasensitive, di-carboxylic acids

## Abstract

Hydrogen-bonded organic frameworks (HOFs), as a newly developed porous material, have been widely used in various fields. To date, several organic building units (OBUs) with tri-, tetra-, and hexa-carboxylic acid synthons have been applied to synthesize HOFs. To our knowledge, di-carboxylic acids have rarely been reported for the construction of HOFs, in particular, di-carboxylic acid-based HOFs with fluorescence sensing properties have not been reported. In this study, a rare example of a di-carboxylic acid-based, luminescent three-dimensional hydrogen-bonded organic framework has been successfully constructed and structurally characterized; it has a strong electron-rich property originated from its organic linker 9-phenylcarbazole-3,6-dicarboxylic acid. It represents the first example of HOF-based sensors for the highly selective and sensitive detection of PA (Picric acid) with reusability; the LOD is less than 60 nM. This work thus provides a new avenue for the fabrication of fluorescent HOFs sensing towards explosives.

## 1. Introduction

Hydrogen-bonded organic frameworks (HOFs), self-assembled by discrete organic molecules via intermolecular hydrogen-bonding interactions, can be further reinforced via weak connections, such as π-π, van der Waals, and/or C-H-π interactions, which has attracted much attention in recent years [1,2,3,4,5,6]. Attributed with some distinct advantages such as orderly pore structures, easy synthesis, facile renewability and recyclability, this kind of crystal materials is investigated in the fields of gas storage and separation [7,8,9], chemical sensing [10,11,12,13,14,15,16], biomedicine [17,18], catalysis [19,20,21], proton conduction [22,23], and so on [24,25,26]. To date, although several organic building units (OBUs) with carboxylic acid synthons have been applied to synthesize HOF, most building units still focus on tri-, tetra-, and hexa-carboxylic acid-building units [27,28,29]. To our knowledge, di-carboxylic acids have rarely been reported for the construction of HOFs [30]. In addition, the aromatic-rich linkers featuring carboxylic acid motifs are more promising in the construction of HOFs with higher resistance to water/solvents owing to the formation of firm H-bonding interactions and strong packing through π-π interactions.

The compound 9-phenylcarbazole has attracted considerable interest as a building block in material science that exhibits an excellent electron-donating ability, well-known hole-transporting properties, photochemical stability, and a rigid structure [31,32,33]. With that, its use for the construction of conjugated HOFs will possess excellent properties and advanced architectures. Therefore, a novel fluorescent hydrogen-bonded organic framework HOF-1 was synthesized by introducing 9-phenylcarbazole-3,6-dicarboxylic acid (H_2_PDA) as the OBUs and excellent fluorescent groups. It can be inferred that the 9-phenylcarbazole part located around the framework of the HOFs endows the HOFs with rich electronic and fluorescence characteristics. The new HOFs can be used for the fluorescence detection of picric acid (PA) molecules. Picric acid is an electron-deficient compound that was widely used as explosives until World War I [34,35]. Due to the fact that explosive materials are increasingly used in industrial and environmental security control, military action security, and terrorism, PA detection by cheap and simple means is an international concern. However, so far, there is no report based on the HOF for PA detection.

## 2. Experiments and Materials

### Synthesis of HOF-1

In a screw-capped vial, a dimethylformamide/water (5/1, 1 mL) solution containing H_2_PDA [36] (3.3 mg, 0.01 mmol) is first formed. Then, the solution is placed into an oven and heated at 140 °C for two days. Needle-like light yellow crystals are generated. Yield: 89%. Anal calc. for C_50_H_32.5_N_2.5_O_10_: C 72.5, H 3.95, N 4.23%; Found: C 72.6, H 3.94, N 4.22%. CCDC number: 2170638 (Tables S1 and S2).

## 3. Results and Discussion

### 3.1. Characterization of HOF-1

**Figure 1:**
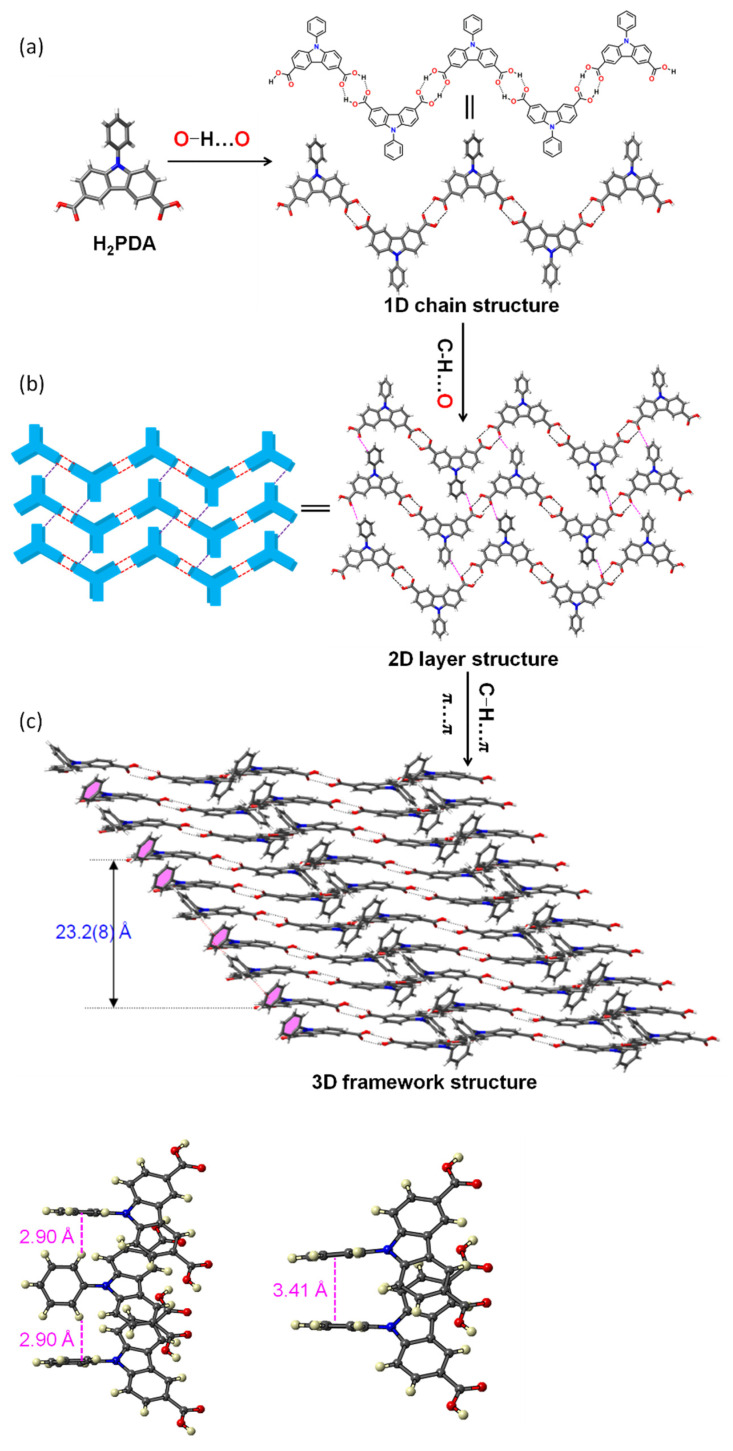
Chemical structures of the fundamental units and related HOF frameworks of HOF-1. (**a**) A 1D Z-shaped chain formed by carboxylic acid dimers of H2PDA through hydrogen bonding interaction. The O–H…O distance is 1.792 Å. (**b**) A two-dimensional (2D) layer formed by two neighboring chains through hydrogen bonding interactions. (**c**) A three-dimensional (3D) framework formed by the neighboring layers in an AABAB fashion through C–H⋯π and π⋯π interactions.

The ligand H_2_PDA was readily synthesized on a gram scale through the Friedel–Crafts acylation reaction followed by a haloform reaction. Needle-shaped light yellow single crystals of HOF-1 ({(H_2_PDA)_2.5_}_n_), large enough for single X-ray diffraction measurement, can be obtained through the solvothermal reaction of H_2_PDA in dimethylformamide/water in a high yield (89%). The monoclinic space group *C*2/*c* exists in HOF-1 through single-crystal X-ray diffraction data. Each molecule links two adjacent molecules via two pairs of intermolecular hydrogen bonds of carboxylic acid dimers. The O⋯O distance was 2.605 Å, and the O–H⋯O angles were 174.22° and 176.50°, respectively. According to the literature [37], they belong to the strong hydrogen bond interaction (distances: 2.49 ~ 3.15 Å). Meanwhile, the distance of O–H⋯O is 1.792 Å, which also corresponds to a strong hydrogen bond interaction (Figure 1a and Figure S1). The connection of these carboxylic acid dimers of H_2_PDA produce a 1D Z-shaped chain. The chain connects with two neighboring chains through a hydrogen bonding interaction (distance 2.64 Å and 2.69 Å) between the phenyl hydrogen atom and the carboxylic oxygen atom of H_2_PDA belonging to neighboring chains, extending into a two-dimensional (2D) layer (Figure 1b and Figure S1). These layers are further interconnected in an AABAB fashion to form a three-dimensional (3D) framework through π⋯π interactions (distance 3.41 Å) between the phenylcarbazole ring belonging to neighboring layers and the C–H⋯π interactions (distance 2.90 Å) between phenylcarbazole hydrogen atom and the neighboring phenylcarbazole rings from different layers (Figure 1c). The closest distance between parallel molecules from the same repeating unit (i.e., the distance between two parallel phenyl rings in the two neighboring AABAB layers) was 23.2(8) Å along the crystallographic b axis. Topologically, the overall structure can be considered to be 1,2,3-connected nodes to form a three-dimensional network with the point symbol of {4^2^6^3^}. HOF-1 is a dense structure, and thus, it was observed without solvent molecules.

**Figure 2:**
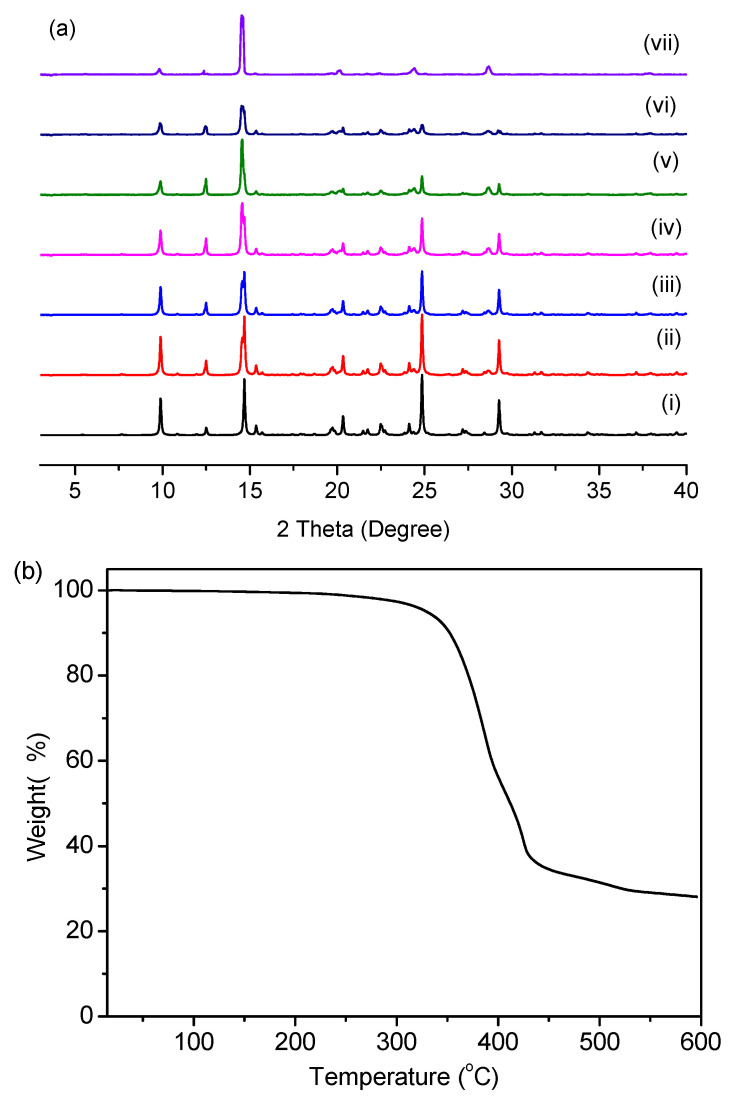
(**a**) Powder X-ray diffraction (PXRD) patterns of simulated HOF-1 (i), as-synthesized (ii), after the treatment with EtOH for one week (iii), after five cycles sensing of PA (iv), after treatment at 100 °C (v), after treatment at 200 °C (vi), and after treatment at 250 °C (vii); (**b**) TGA traces of HOF-1 ranging from room temperature to 600 °C.

Powder X-ray analysis showed there is consistency between the patterns of as-synthesized HOF-1 and the simulated one, indicating that the HOF-1 samples were pure. The N_2_ isotherm shows a reversible-type IV behavior with a calculated Brunauer−Emmett−Teller (BET) surface area of 18 m^2^ g^−1^. The pore-size distribution of HOF-1 according to the NLDFT model indicates a maximum at 0.8 nm (Figures S2 and S3). Further evidence on the robustness of HOF-1 comes from the excellent thermal stability supported by the thermogravimetric analysis, showing barely a reduction in weight from 25 °C to 330 °C; thus this plateau can be maintained until HOF-1 starts decomposition at >330 °C (Figure 2b). To probe the structural stability, we also performed a variable-temperature PXRD study of HOF-1; the results, shown in Figure 2a, suggest that the crystal phase does not change until at least 200 °C. To investigate the chemical stability of HOF-1 in ethanol (EtOH), powder XRD patterns of the HOF-1 samples immersed in EtOH for one week were tested; the results evidence the maintenance of the framework (Figure 2a). It is its intensive structure character that may lead to its good structural stability and thermal stability.

### 3.2. Sensing of PA

**Figure 3:**
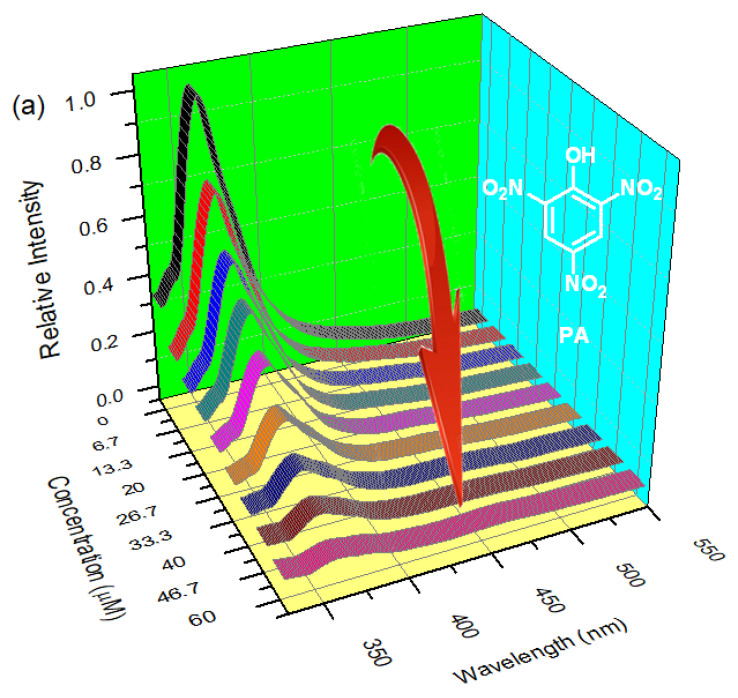

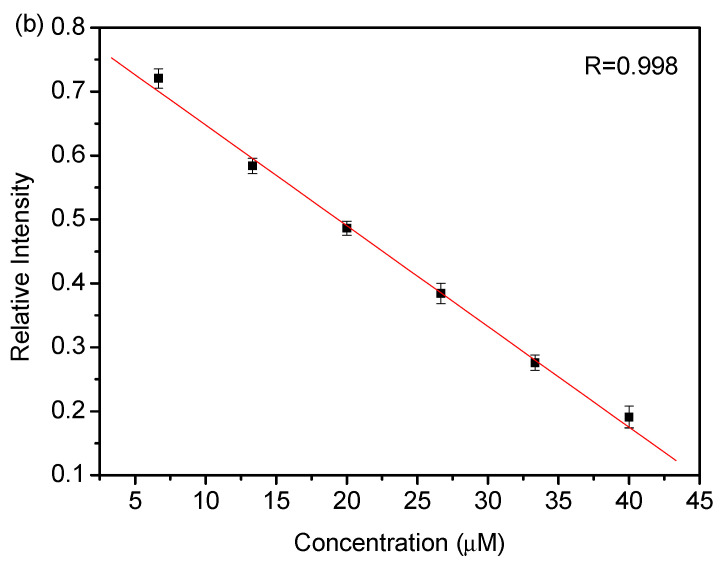
(**a**) The fluorescence titration data of an EtOH solution of PA in the suspension of HOF-1. (**b**) The linear relationship between the fluorescence intensity of HOF-1 and the PA concentration in the range of 5−40 μM.

The π-electron-rich environment in the entire HOF framework stimulated us to investigate the sensing behavior of HOF-1 towards aromatic compounds. When excitation at 320 nm occurs, HOF-1 with a concentration of 0.33 g L^−1^ in EtOH showed an intense luminescence band at about 360 nm. Moreover, the emission spectra of HOF-1 after being stored in EtOH for one week was measured (Figure S4), and the luminescent intensity of the treated HOF-1 did not almost change. In addition, the pH-dependent fluorescence response of HOF-1 was also evaluated (Figure S5), the emission intensity of HOF-1 remained almost unchanged in the pH range of 5.5−9.3, all suggesting its applicability as a fluorescent sensor in ethanol or aqueous environments. Since the nitrated aromatic explosives is soluble in ethanol, the properties of the material as a fluorescent probe in EtOH solution were investigated. In order to explore the luminescence response of HOF-1 to PA, the suspension with the concentration of 0.33 g L^−1^ was generated by ultrasonic wave through dispersing the ground sample in ethanol solution. As shown in Figure 3a, when the ethanol solution of PA is gradually added to HOF-1 suspension, the emission intensity decreases significantly. The fluorescence intensity of 94% was quenched when PA at a concentration of 60 μM was added to the HOF-1 EtOH emulsion. As a comparation, adding PA (60 μM) to the free H_2_PDA ethanol solution caused only a less than 7% intensity decrease of the luminescence at 360 nm of the ligand (Figure S6). Therefore, HOF-1 presents a characteristic property of PA detection in a luminescence-quenching manner, demonstrating that the crystalline structure of HOF-1 could contribute in sensing of PA.

The detection limit of HOF-1 for PA is less than 60 nM according to the 3δ IUPAC standard, which shows that HOF-1 can be used as a turn-off fluorescent probe material with excellent sensitivity. In addition, the fluorescence intensity of HOF-1 has a good linear relationship with PA in the concentration range of 5−40 μM, which indicates that HOF-1 has the potential to detect PA quantitatively (Figure 3b). The Stern−Volmer equation, the expression of which is I_0_/I = 1 + *K*_SV_[M], could be used to process the data of the luminescence titration, where *K*_SV_ represents the Stern−Volmer constant, I represents the luminescence intensity upon PA adding, I_0_ represents HOF-1’s original luminescence intensity, and [M] represents PA molar concentration. On the basis of the luminescence titration data in Figure 3a and the above equation, the *K*_SV_ value was 5.9 × 10^5^ M^−1^ based on that calculation (Figure S7); this value is comparable to those of the MOFs- and COFs-based sensors for PA determination in solution (Table S3).

**Figure 4:**
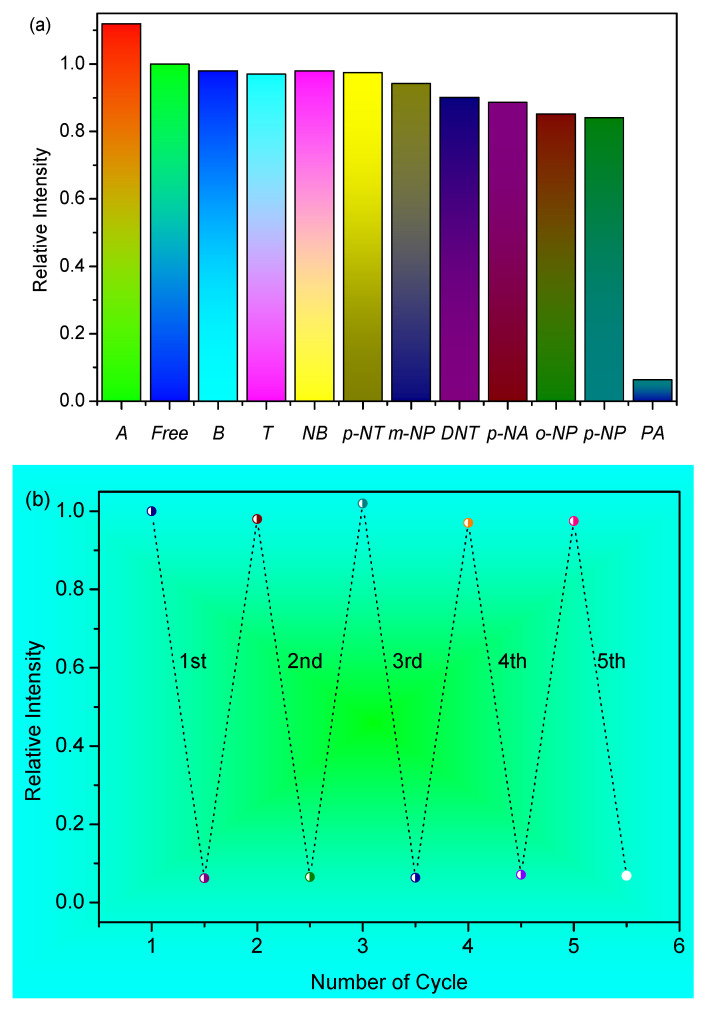
(**a**) The quenching efficiency of HOF-1 upon addition of different aromatic compounds. (**b**) The fluorescence recovery and fluorescence detection effect of re-generated HOF-1 washing five times with ethanol.

To investigate whether HOF-1 displayed the sensing of PA with good selectivity, luminescent detection tests were carried out with other aromatic compounds, including nitrobenzene (NB), benzene (B), toluene (T), aniline (A), 2,4-dinitrotoluene (DNT), *p*-nitrotoluene (*p*-NT), *p*-nitroaniline (*p*-NA), *p*-nitrophenol (*p*-NP), *m*-nitrophenol (*m*-NP), and *o*-nitrophenol (*o*-NP). As shown in Figure 4a and Figure S8, the presence of *p*-NT, NB, T, and B hardly changes the spectra of HOF-1. The addition of aniline led to a slight increase in fluorescence. The addition of *m*-NP, DNT, *p*-NA, *o*-NP, and *p*-NP to the HOF-1 ethanol solution caused ca. 5.8%, 9.9%, 11.4%, 14.8%, and 15.9% quenching of the fluorescence, respectively. Compared with other aromatic compounds, only the addition of PA to the HOF-1 ethanol suspension led to a ca. 94% quenching of the fluorescence, indicating that HOF-1 has a remarkable selectivity for PA. As far as we are concerned, HOF-1 represents the first HOF-based chemosensor for PA with such a high sensitivity and selectivity.

As a PA sensor, the regeneration is extremely important for practical applications. Therefore, we also investigated the luminescence properties of the re-generated HOF-1. The previously used HOF-1 that was washed five times with ethanol to obtain the regenerated HOF-1 material. It is worth noting that the emission intensity of the regenerated HOF-1 almost recovered, and a similar fluorescence response was observed for PA (Figure 4b). Within five cycles, the initial fluorescence intensity of the HOF-1 almost completely recovered (about 97.5%), which means that they have high photostability, so they may be suitable for long-term detection for PA or applications in environmental monitoring. Meanwhile, the PXRD data also show the integrity of the HOF-1’s framework structure (Figure 2a). Moreover, the FT-IR spectra show that the vibration bands of HOF-1 after the fifth repetitive run matched that of the original one well, further confirming the stability of the HOF material during PA sensing (Figure S9). Therefore, these results reveal that the luminescent quenching of HOF-1 did not originate from the collapse of the framework. The UV–Vis absorption bands of PA and H_2_PDA partly overlap each other (Figure S10), and the competition for the irradiated light absorption between the PA and the ligand is one of the reasons that lead to fluorescence quenching. Considering that there is a large difference between the quenching degree of the H_2_PDA ligand and the HOF-1 caused by PA, the mechanism of detection of HOF-1 against PA may be attributed to the synergies of competitive absorption among PA and HOF-1 and the framework structure of HOF-1 itself.

## 4. Conclusions

In conclusion, we successfully created a rare example of a di-carboxylic acid-based HOF by incorporating the 9-phenylcarbazole moiety as an emission unit, which exhibits excellent ethanol and thermal stability. HOF-1 is the first example of HOFs as fluorescent sensors for PA-efficient detection with LOD of 60 nM. Additionally, the linear response result with the PA concentration from 5 to 40 μM enables HOF-1 to quantitatively detect PA. It is noteworthy that HOF-1 keeps its original luminescent reactivity during prolonged sensing recycling for at least five cycles. This work not only plays a leading role in the synthesis of HOFs, but also shows the ideal effect in detecting dangerous goods and meets sustainable requirements in the industry.

## Data Availability

All data are contained within the article.

## Supplementary Materials

The following supporting information can be downloaded at: https://www.mdpi.com/article/10.3390/bios12090682/s1, Figure S1: A 1D Z-shaped chain formed by carboxylic acid dimers of H2PDA; Figure S2: N2 physisorption isotherms of HOF-1; Figure S3: Pore size distribution of HOF-1; Figure S4: Luminescence spectra of HOF-1 before (black) and after (red) storage in ethanol for one week; Figure S5: pH-dependent fluorescence of HOF-1 in aqueous with the pH ranging from 5.5 to 9.3; Figure S6: Fluorescence spectra of H2PDA in ethanol solution upon the addition of 60 μM of PA; Figure S7: The Stern−Volmer plot of HOF-1 quenched by PA, where I0 and I are the fluorescence intensity before and after PA incorporation, respectively; Figure S8: Families of various fluorescence spectra of HOF-1 in ethanol solution upon the addition of 60 μM of different selected aromatic compounds; Figure S9: FT-IR spectra of original HOF-1 and HOF-1 after the 5th PA sensing; Figure S10: UV-vis spectra of PA and HOF-1; Table S1: Atom sites for HOF-1; Table S2: Crystallographic data for HOF-1; Table S3: Comparison with different MOFs/COFs sensors for PA detection.
