# Supplementary material for: An Ultrasensitive Picric Acid Sensor Based on a Robust 3D Hydrogen-Bonded Organic Framework
Source: Biosensors (Basel). 2022 Aug 25;12(9):682. doi: 10.3390/bios12090682 (PMC9496322; doi:10.3390/bios12090682)
Supplement: Supplementary file 1 [file biosensors-12-00682-s001.zip › biosensors-1858805-supplementary.pdf]

Supporting Information for

An Ultrasensitive Picric Acid Sensor Based on a Robust 3D Fluorescent Hydrogen-Bonded Organic Framework

Weiwei Jiang,[1] Lingling Xia,[1] Dan Li,[1] Pengyan Wu,*[1] Tongtong Zou,[1] Xingcheng Yuan,*[1] Wen Wei[1] and Jian Wang*[1]

[1] *School of Chemistry and Materials Science & Jiangsu Key Laboratory of Green Synthetic Chemistry for Functional Materials, Jiangsu Normal University, Xuzhou, Jiangsu, 221116, P. R. China; E-mail: wpyan@jsnu.edu.cn; xchyuan@jsnu.edu.cn; wjian@jsnu.edu.cn*

Materials and Methods.

All chemicals were purchased commercially and were not purified, which all have reagent grade quality. 9-Phenylcarbazole (98%) was purchased from Qingdao Frontierchem Co., Ltd., Picric Acid and the other nitroaromatics were provided from Xiya Reagent Company (China). A Vario EL III elemental analyzer was used to examine C, H and N elemental analyses. A Mettler-Toledo TGA/SDTA851 instrument was used to test thermogravimetric analysis (TGA) in a nitrogen flow at a 5 °C/min ramp rate. Mass spectra were carried out on a Bruker micro TOF mass spectrometer. A Rigaku D/max-2400 X−ray powder diffractometer was used to test X-Ray powder diffraction (XRD) patterns of the HOF-1 using Cu Ka (λ =1.5405 Å) radiation, and the variable-temperature PXRD study of HOF-1 was heated HOF-1 at 373 K, 473 K and 523 K for 6 h under vacuum, respectively. A Bruker-400 spectrometer was used to test ^{1}H NMR spectra with Me_4Si as an internal standard. Solid UV-visible adsorption spectra were measured with a SHIMADZU UV-2600 spectrometer. N_2 adsorption-desorption isotherms were measured at 77 K using a Quantachrome autosorb iQ2 analyzer, and surface areas of samples were calculated by the Brunauer-Emmett-Teller (BET) method and pore size distributions were determined by the nonlocalized density functional theory (NLDFT) model. Hitachi F-4500 instrument was used to measure the solution fluorescent spectra. 2.5 nm was chosen as emission and excitation slit widths. Test samples and recorded method were prepared as follows: HOF-1 powder: 1 mg, ethanol: 3.00 mL, uniform dispersing; recorded intensity: 360 nm, excitation: 320 nm. Guest samples were prepared as follows: related nitro-analysts: 2.0×10^{-2} M, solvent: ethanol.

Crystallography:

Intensities were collected on a Bruker SMART APEX CCD diffractometer with graphitemonochromated Mo-Kα (λ = 0.71073 Å) using the SMART and SAINT programs. The structure was solved by direct methods and refined on F^2 by full-matrix least-squares methods with SHELXTL *version* 5.1. Non-hydrogen atoms of the ligand backbones were refined anisotropically. Hydrogen atoms within the ligand backbones were fixed geometrically at calculated positions and allowed to ride on the parent non-hydrogen atoms.

Figure S1. A 1D Z-shaped chain formed by carboxylic acid dimers of H_2PDA through hydrogen bonding interaction (top) and a two-dimensional (2D) layer formed by two neighboring chains through hydrogen bonding interaction (bottom). .

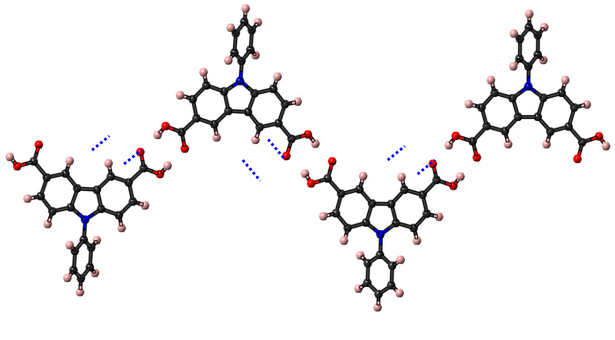

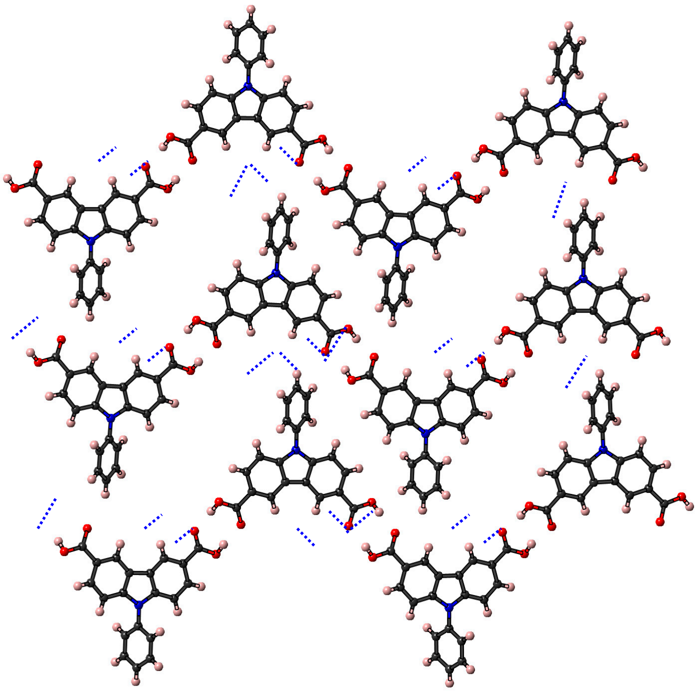

Table S1. Atom sites for HOF-1.

Atom site label	Atom site fract x	Atom site fract y	Atom site fract z
O3	-0.03644(7)	0.30055(7)	0.51883(11)
O15	0.23314(8)	0.32604(8)	-0.01176(12)
O16	0.20825(9)	0.25540(9)	0.08164(13)
O17	-0.01122(7)	0.23739(7)	0.41375(12)
O8	0.33598(7)	0.14650(8)	0.47956(11)
O12	0.30247(8)	0.21492(8)	0.56684(13)
O13	0.07262(8)	0.15504(8)	1.01812(12)
O14	0.09898(8)	0.22014(8)	0.91720(13)
O1	0.10894(7)	0.26452(7)	0.58579(12)
O2	0.14044(7)	0.19733(7)	0.49296(11)
C26	0.12308(9)	0.43224(10)	0.20412(14)
C28	0.03273(9)	0.43900(9)	0.39011(15)
C29	0.14238(9)	0.33189(10)	0.17212(15)
C41	0.07893(9)	0.36917(9)	0.29600(14)
C43	0.01914(9)	0.33523(9)	0.40695(14)
C60	0.18299(9)	0.40943(11)	0.08851(15)
C75	0.20745(10)	0.30656(11)	0.05649(15)
C78	0.05428(9)	0.32320(9)	0.33829(14)
C82	0.10548(9)	0.55477(10)	0.35292(15)
C88	0.09057(8)	0.52604(10)	0.26771(15)
C92	0.00901(9)	0.39282(10)	0.43209(15)
C94	0.07080(10)	0.61751(10)	0.18880(17)
C97	0.07326(9)	0.55752(10)	0.18546(16)
C105	0.10262(10)	0.61481(10)	0.35561(17)
C107	0.17608(9)	0.35038(10)	0.10590(15)
C111	0.15652(9)	0.45141(10)	0.13725(15)
C112	0.08542(10)	0.64616(11)	0.27442(17)
C113	0.06725(9)	0.42649(9)	0.32065(14)

C115	0.11533(9)	0.37278(9)	0.22182(14)
C120	-0.01084(9)	0.28747(10)	0.44818(15)
C10	0.17686(9)	0.03422(10)	0.81031(16)
C19	0.16069(9)	0.13681(10)	0.83207(15)
C38	0.12954(9)	0.12294(10)	0.90503(16)
C40	0.21860(9)	0.09090(10)	0.70846(15)
C48	0.25706(9)	0.01603(10)	0.61452(16)
C52	0.09931(10)	0.16925(10)	0.94893(16)
C54	0.28056(9)	0.05927(10)	0.56559(16)
C61	0.27470(9)	0.11818(10)	0.58841(15)
C76	0.25728(10)	-0.09243(10)	0.76358(18)
C79	0.24249(9)	0.13435(10)	0.65862(15)
C80	0.20413(9)	-0.06401(10)	0.75579(15)
C87	0.12482(10)	0.06468(10)	0.93241(17)
C100	0.18481(9)	0.09242(10)	0.78576(15)
C108	0.22749(9)	0.03253(10)	0.68806(16)
C109	0.14814(10)	0.01990(11)	0.88687(17)
C116	0.30502(9)	0.16421(10)	0.54302(15)
C119	0.20818(11)	-0.18343(12)	0.76995(19)
C122	0.15619(12)	-0.15483(11)	0.7609(2)
C123	0.15331(11)	-0.09494(11)	0.7527(2)
C129	0.25884(11)	-0.15228(11)	0.76989(19)
C12	0.02880(9)	0.07939(9)	0.69247(14)
C13	0.01842(9)	0.13786(9)	0.71366(14)
C16	0.000000	-0.01728(13)	0.750000
C18	0.06404(9)	0.06403(9)	0.62601(15)
C23	0.04484(8)	0.18176(9)	0.66836(14)
C33	0.11128(9)	0.21383(10)	0.55914(15)
C34	0.08970(9)	0.10827(10)	0.58122(15)
C37	0.08036(9)	0.16690(9)	0.60256(14)
C51	-0.01292(9)	-0.10764(10)	0.66553(16)
C63	0.000000	-0.13752(14)	0.750000
C69	-0.01311(9)	-0.04755(9)	0.66520(15)
N1	0.09359(7)	0.46471(8)	0.26455(12)
N4	0.20221(7)	-0.00178(9)	0.75110(12)
N2	0.000000	0.04418(11)	0.750000
H9A	-0.054847	0.272971	0.538878
H15A	0.251768	0.298874	-0.032463
H28A	0.025882	0.477852	0.407702
H29A	0.137815	0.291654	0.183406
H60A	0.206230	0.421078	0.042494
H78A	0.061166	0.284324	0.320898
H82A	0.117591	0.533447	0.409226
H92A	-0.014831	0.400110	0.479306
H94A	0.059127	0.639033	0.132551
H97A	0.063117	0.537880	0.126948
H10A	0.112645	0.634510	0.414097
H11A	0.161061	0.491574	0.125403
H11B	0.083557	0.687290	0.276937
H8A	0.352654	0.175727	0.461007
H20A	0.055994	0.184459	1.031694
H19A	0.165285	0.176084	0.814449
H48A	0.260885	-0.023656	0.598652
H54A	0.301176	0.049143	0.515356
H76A	0.292186	-0.070951	0.764559
H79A	0.236901	0.174112	0.672266

H87A	0.104852	0.056035	0.984065
H10B	0.144953	-0.019206	0.906432
H11C	0.209439	-0.224450	0.776231
H12A	0.121377	-0.176422	0.760232
H12B	0.116900	-0.075584	0.745023
H12C	0.294923	-0.172060	0.774211
H2A	0.158785	0.225467	0.474539
H18A	0.070340	0.024491	0.611760
H23A	0.038565	0.221330	0.682416
H34A	0.113953	0.098941	0.535441
H51A	-0.021652	-0.128363	0.607750
H63A	0.000000	-0.178733	0.750000
H69A	-0.022159	-0.027023	0.607286

Table S2. Crystallographic data for HOF-1.

	**HOF-1**
formula	$C_{50}H_{32.5}N_{2.5}O_{10}$
formula weight	828.28
crystal system	monoclinic
space group	*C*2/*c*
a /Å	23.284(3)
b /Å	23.052(3)
c /Å	14.2985(16)
β (°)	98.468(2)
V /Å^3	7590.9(16)
Z	8
ρ_{calcd}/g cm^{-3}	1.450
μ /mm^{-1}	0.102
Collected reflections	6667
unique reflections	5184
R_1 [$I > 2\sigma(I)$]	0.0736
wR_2 (all data)	0.2079

Figure S2. N_2 physisorption isotherms of HOF-1.

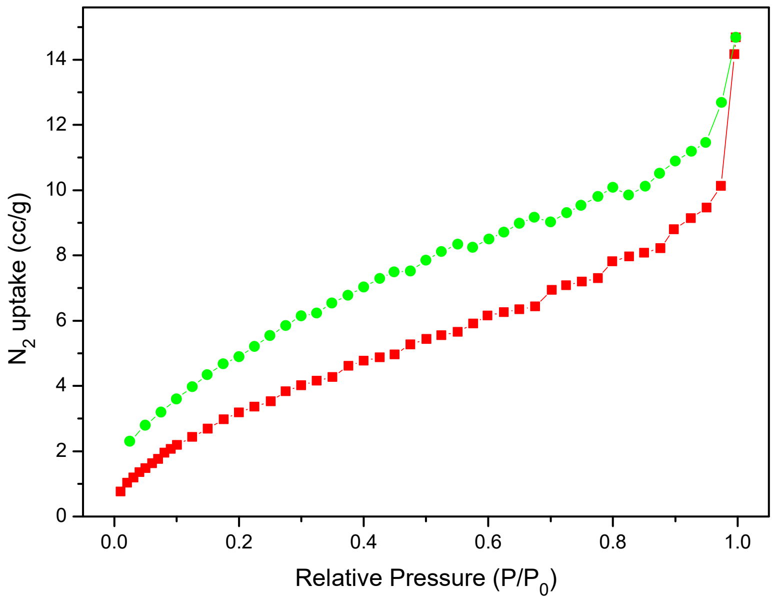

Figure S3. Pore size distribution of HOF-1.

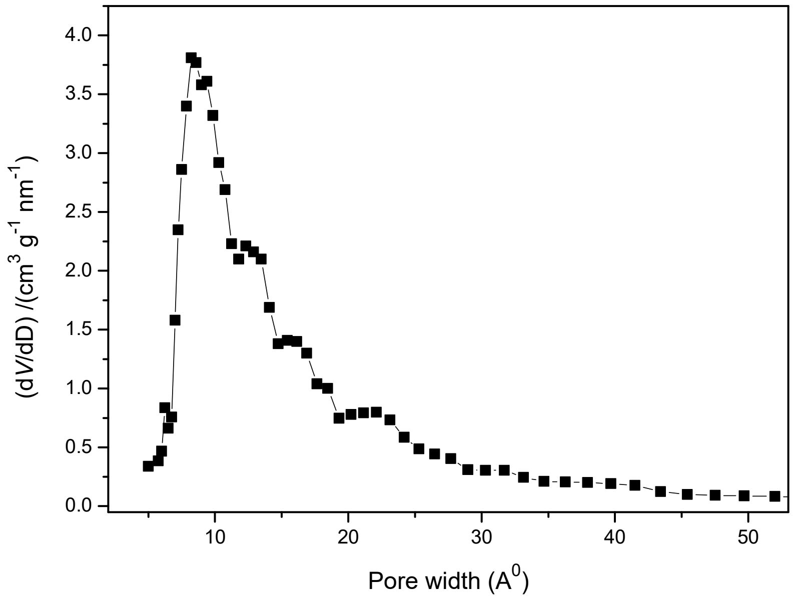

Figure S4. Luminescence spectra of HOF-1 before (black) and after (red) storage in ethanol for one week.

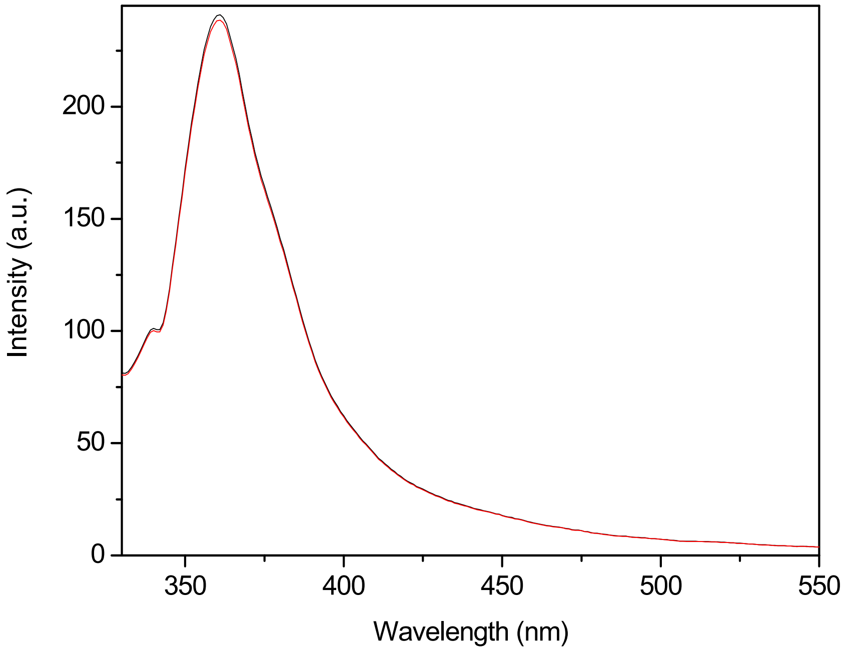

Figure S5. pH-dependent fluorescence of HOF-1 in aqueous with the pH ranging from 5.5 to 9.3.

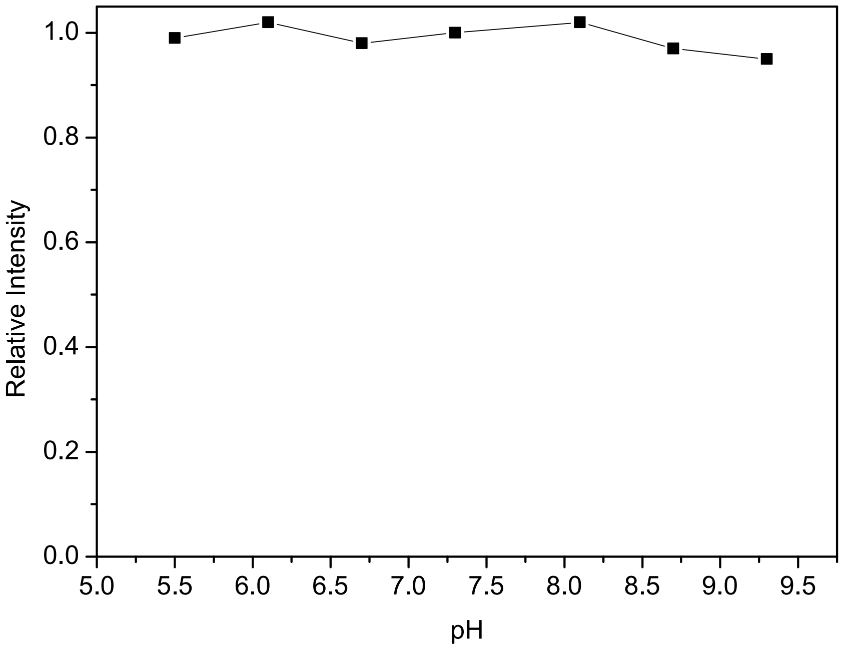

Figure S6. Fluorescence spectra of H_2PDA in ethanol solution upon the addition of 60 μM of PA.

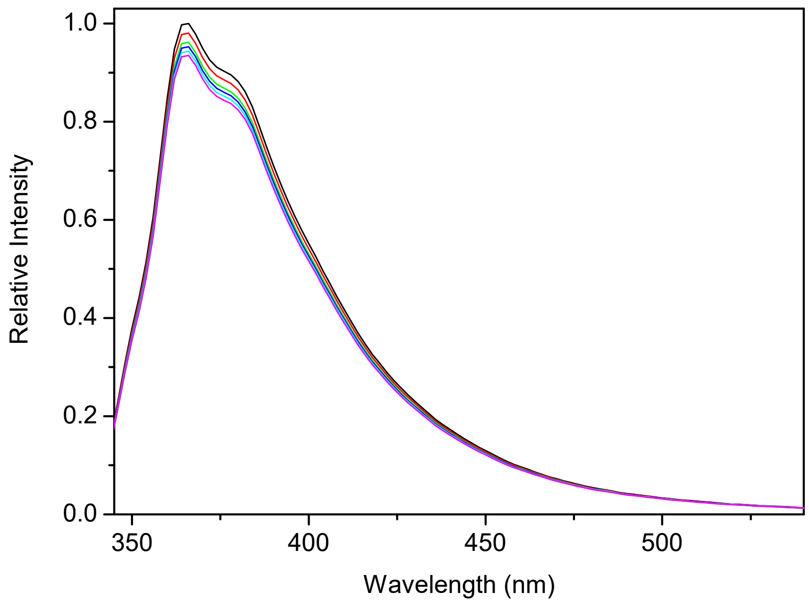

Figure S7. The Stern−Volmer plot of HOF-1 quenched by PA, where I_0 and I are the fluorescence intensity before and after PA incorporation, respectively.

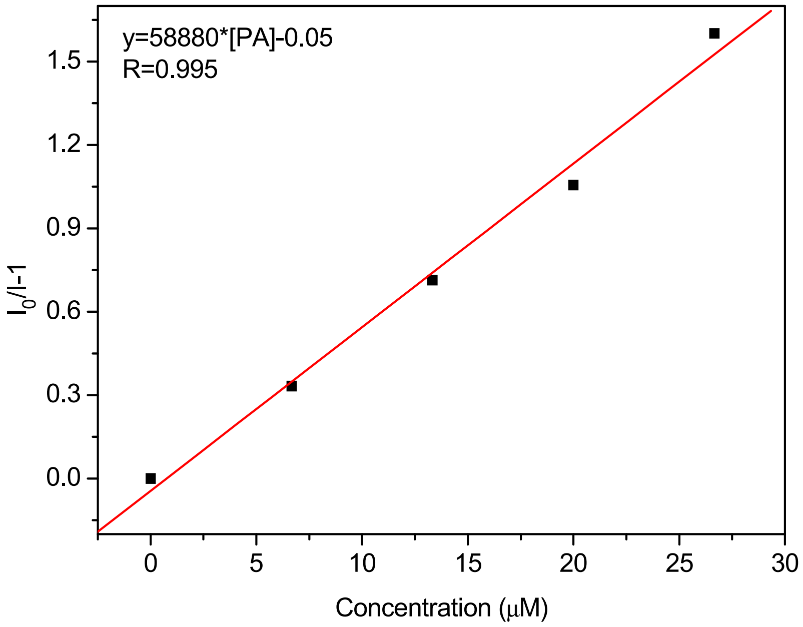

Figure S8. Families of various fluorescence spectra of HOF-1 in ethanol solution upon the addition of 60 μM of different selected aromatic compounds.

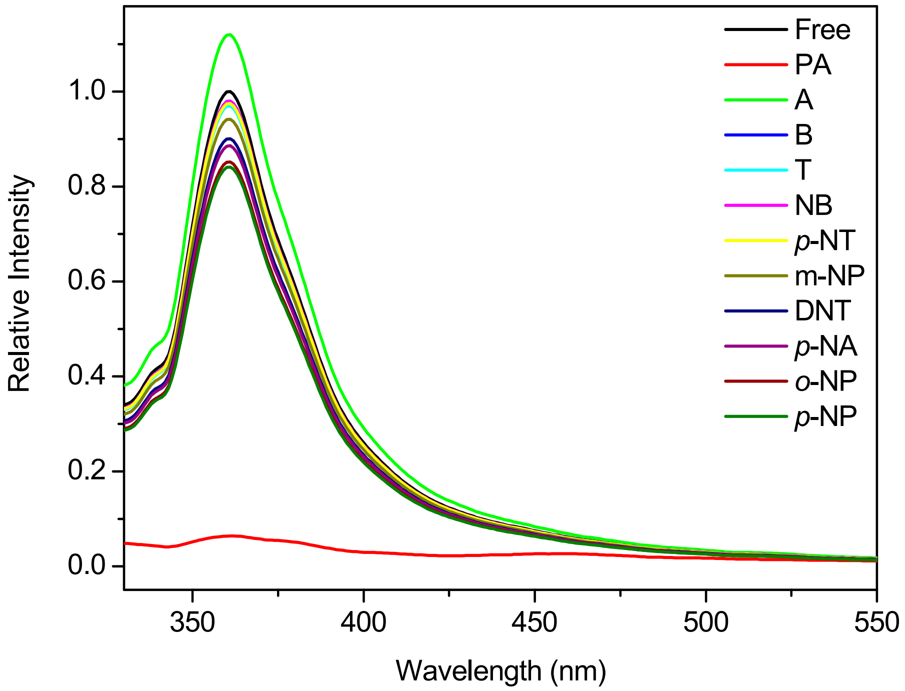

Figure S9. FT-IR spectra of original HOF-1 and HOF-1 after the 5th PA sensing.

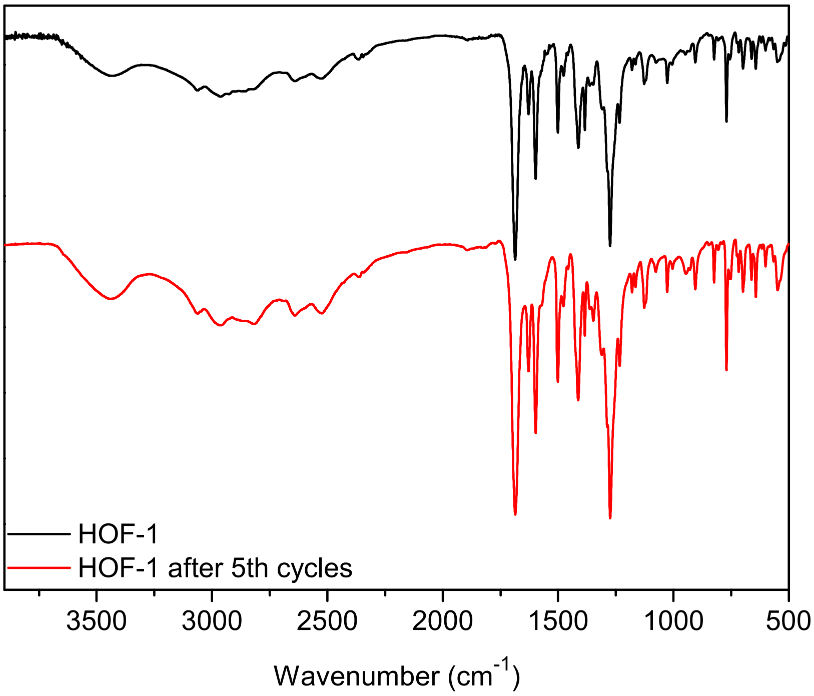

Figure S10. UV-vis spectra of PA and HOF-1.

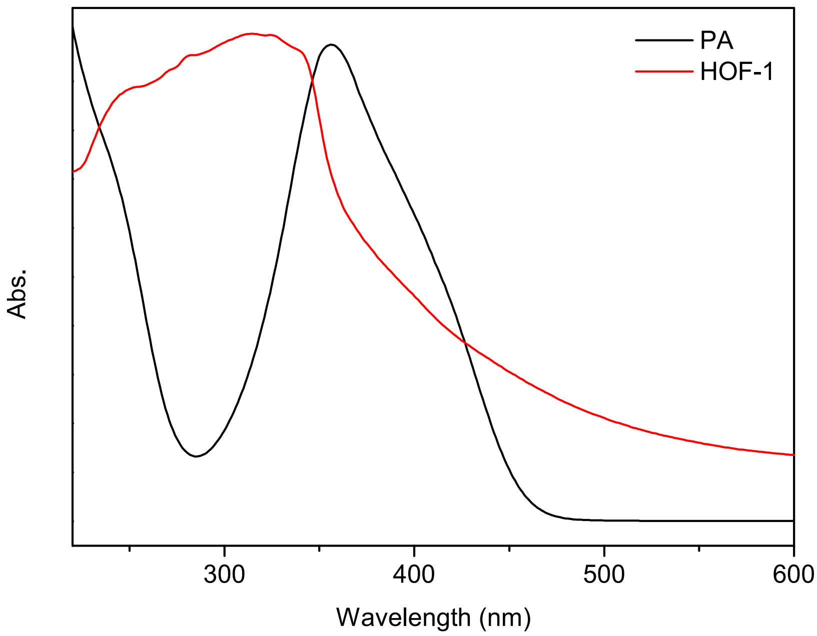

Table S3. Comparison with different MOFs/COFs sensors for PA detection.

Entry	Materials	LOD	*K*sv (M^{-1})	Ref.
1	DTZ-COF	0.36 μM	8.71×10^4	[1]
2	PTPATCz	2.99 nM	1.00×10^3	[2]
3	SNW-1	0.05 μM	9.4×10^4	[3]
4	TTRZ	27 ppb	1.15×10^5	[4]
5	$[(CH_3)_2NH_2]_3[Zn_4Na(BPTC)_3]\cdot 4MeOH\cdot 2DMF$	5 μM	3.2×10^4	[5]
6	$[Cd_2(PAM)_2(dpe)_2(H_2O)_2]\cdot 0.5dpe$	7.4 nM	7.47×10^4	[6]
7	$[Zn_8(ad)_4(BPDC)_6O_2Me_2NH_2]$	12.9 nM	4.6×10^4	[7]
8	$[Zr_6O_4(OH)_4(L)_6]$	1.7 μM	5.8×10^4	[8]
9	$[Zn_4(DMF)(Ur)_2(NDC)_4]$	7.1 μM	10.83×10^4	[9]
10	$[Zn_2(TPOM)(NH_2\text{-}bdc)_2]\cdot 4H_2O$	0.98 μM	4.6×10^4	[10]
11	$[Zn_2(L)_2(bpy)]\cdot DMF\cdot 2H_2O$	3.48 μM	1.53×10^4	[11]
12	$[Zn_2(L)_2(azp)]\cdot 2DMF\cdot H_2O$	1.82 μM	3.11×10^4	
13	$[Eu_2(L)_2(OH)(HCOO)]\cdot H_2O$	2.71 μM	1.21×10^4	[12]
14	$[Tb_2(L)_2(OH)(HCOO)]\cdot H_2O$	2.35 μM	1.17×10^4	
15	$[Eu_4(NDC)_6(H_2O)_5]\cdot 3H_2O$	0.67 μM	2.6×10^4	[13]
16	$[Cd_3(SDB)_3(TIB)](H_2O)_2$(1,4-dioxane)	0.15 μM	2.43×10^4	[14]
17	[Zn(IPA)(L)]	0.12 μM	2.16×10^4	[15]
18	[Zn(IPA)(L)]n	0.06 μM	1.52×10^4	
19	$[Zn(NH_2\text{-}bdc)(4,4'\text{-}bpy)]$	0.32 μM	3.04×10^4	[16]
20	$\{[W_2(\mu_3\text{-}S)_6(\mu_4\text{-}S)_2Cu_8(CN)_4(3\text{-}abpt)]\cdot MeCN\cdot H_2O\}_n$	0.81 μM	7.66×10^4	[17]
21	CSMCRI-9	66 nM	2.46×10^4	[18]
22	ZSTU-2	2.37 μM	2.27×10^4	[19]
23	CSMCRI-6	67 nM	2.27×10^5	[20]
24	$\{Mn(Tipp)(A)_2\}_n\cdot 2H_2O$	0.2 μM	1.18×10^5	[21]
25	$Pb_7(TTPCA)_4Cl_2]\cdot 3H_2O$	1.03 μM	5.22×10^4	[22]
26	$[Pb_7(TTPCA)_4(DMA)_2(HCOO)_2]\cdot H_2O$	1.03 μM	4.33×10^4	
27	$[Pb_4(TTPCA)_3]\cdot 3DMF\cdot 2H_2O\cdot H_3O$	2.63 μM	3.14×10^4	
28	$[(Zn_4O)(H_2O)_2(TPA)_2]\cdot 8DMA$	0.6 μM	1.14×10^5	[23]
29	H_8L-Ti-MOF	3.6 μM	8.7×10^5	[24]
30	$[Zn_3(btc)_2(tpt)(H_2O)_2]\cdot 4H_2O$	0.94 μM	8.42×10^4	[25]
31	Zn-MOF	0.17 mM	3.62×10^4	[26]
33	Cu(I)-MOF	1.09 μM	1.5×10^4	[27]
34	$[Cu_2(tpt)_2(tda)_2]\cdot H_2O$	2.71 μM	1.36×10^5	[28]
35	$\{Co(TCPP)_{0.5}(Tipa)\}_n$	0.33 μM	5.5×10^4	[29]
36	$Cu_2I_2(MA)_2$	0.72 μM	2.22×10^5	[30]
37	$[Mg_3L_2(H_2O)_6]_2\cdot 12H_2O$	0.13 μM	2.6×10^4	[31]
38	$[Zn\ (tbda)]_n$	0.26 mM	3.3×10^3	[32]
39	$\{[Dy(\mu_2\text{-}FcDCA)_{1.5}(MeOH)(H_2O)]\cdot 0.5H_2O\}_n$	0.71 μM	8.55×10^4	[33]
40	$[Zn(BPDPE)_2(dca)_2]_n$	0.73 μM	5.3×10^4	[34]
41	$\{[Cd_4(L)_2(oba)_4]\cdot 4H_2O\}_n$	0.67 μM	1.9×10^5	[35]
42	$\{[Co_4(L)_2(BPDC)_4\cdot 4H_2O]\cdot 5MeCN\cdot 5H_2O\}_n$	0.67 μM	4.7×10^5	
43	HOF-1	60 nM	5.9×10^5	This work

Reference

1. Y. Li Y. Han, M. Chen, Y. Feng and B. Zhang, *RSC Adv.* **2019**, *9*, 30937–30942.
2. T. Geng., Z. Zhu, X. Wang, H. Xia, Y. Wang and D. Li, Sens. Actuators B Chem., **2017**, *244*, 334–343.
3. W. Zhang, L.-G. Qiu, Y.-P. Yuan, A.-J. Xie, Y.-H. Shen and J.-F. Zhu, J. Hazard. Mater., **2012**, *221*, 147–154.
4. Y. Zhuang, H. Shan, Z. Zhang, S. Li, Q. Zhu, Z. Si, S. Yang, Z. Yang, D. Cai and P. Qin, Dyes Pigm., **2021**, *192*, 109421.
5. J.-N. Hao and B. Yan, New, J. Chem., **2016**, *40*, 4654–4661.
6. E.-L. Zhou, P. Huang, C. Qin, K.-Z. Shao and Z.-M. Su, J. Mater. Chem. A., **2015**, *3*, 7224–7228.
7. J. Ye, X. Wang, R.F. Bogale, L. Zhao, H. Cheng, W. Gong, J. Zhao and G. Ning, Sens. Actuators B Chem., **2015**, *210*, 566–573.
8. L.-H. Cao, F. Shi, W.-M. Zhang, S.-Q. Zang and T. C. W. Mak, Chem. Eur. J., **2015**, *21*, 15705–15712.
9. B. Joarder, A.V. Desai, P. Samanta, S. Mukherjee and S. K. Ghosh, Chem. Eur. J., **2015**, *21*, 965–969.
10. S. S. Nagarkar, A.V. Desai, P. Samanta and S. K. Ghosh, Dalton Trans., **2015**, *44*, 15175–15180.
11. B.-Q. Song, C. Qin, Y.-T. Zhang, X.-S. Wu, L. Yang, K.-Z. Shao and Z.-M. Su, Dalton Trans., **2015**, *44*, 18386–18394.
12. S. S. Nagarkar, B. Joarder, A.K. Chaudhari, S. Mukherjee and S. K. Ghosh, Angew. Chem. Int. Ed., **2013**, *52*, 2881–2885.
13. R. Lv, J. Wang, Y. Zhang, H. Li, L. Yang, S. Liao, W. Gu and X. Liu, *J. Mater. Chem. A.*, **2016**, *4*, 15494–15500.
14. S. Senthilkumar, R. Goswami, N.L. Obasi and S. Neogi, *ACS Sustain. Chem. Eng.*, **2017**, *5*, 11307–11315.
15. J.-H. Qin, H.-R. Wang, M.-L. Han, X.-H. Chang and L.-F. Ma, *Dalton Trans.*, **2017**, *46*, 15434–15442.
16. T. Wiwasuku, J. Boonmak, K. Siriwong, V. Ervithayasuporn and S. Youngme, Sens. Actuators B Chem., **2019**, *284*, 403–413.
17. J. Zhang, Y. Liu, J. Feng, L. Gong, M.G. Humphrey and C. Zhang, Inorg. Chem., **2019**, *58*, 9749–9755.
18. N. Seal, M. Singh, S. Das, R. Goswami, B. Pathak and S. Neogi, Mater. Chem. Front., **2021**, *5*, 979–994.
19. X. Zhang, Y. Yan, F.Q. Chen, G.X. Bai, H. Xu and S. Q. Xu, Z. Anorg. Allg. Chem., **2021**, *647*, 759–763.
20. R. Goswami, S. Das, N. Seal, B. Pathak and S. Neogi, ACS Appl. Mater. Interfaces., **2021**, *13*, 34012–34026.
21. Y. Zou, K. Huang, X.Y. Zhang, D.B. Qin and B. Zhao, Inorg. Chem., **2021**, *60*, 11222–11230.
22. S. Yu, K.Y. Zhang, J.X. Li, Y. Xiao, L.X. Sun, F.Y. Bai and Y. H. Xing, Inorg. Chem., **2021**, *60*, 7887–7899.
23. W. Xie, W. Jiang, G.J. Xu, S.R. Zhang, Y.H. Xu and Z. M. Su, CrystEngComm., **2021**, *23*, 3901–3906.
24. D. Chakraborty, S. Bej, S. Sahoo, S. Chongdar, A. Ghosh, P. Banerjee and A. Bhaumik, ACS Sustain. Chem. Eng., **2021**, *9*, 14224–14237.
25. Y. Meng, N.X. Zhang, J.X. Li, Y.Y. Xu, Q.F. Yang, Y. Yuan, X. Zhang, J. Wu and L. Y. Zhao, Spectrochim. Acta A Mol. Biomol., **2022**, *266*, 120419.
26. H. Chong, Y.H. Xu, Y. Han, C.G. Yan, D.W. Su and C. Y. Wang, Chemistryselect., **2021**, *6*, 9363–9367.
27. K. Wu, J. Hu, X. Cheng, J. Li and C. Zhou, J. Lumin., **2020**, *219*, 116908.
28. X. R. Zhuang, N.X. Zhang, X. Zhang, Y. Wang, L.Y. Zhao and Q. F. Yang, Microchem. J., **2020**, *153*, 104498.
29. X. L. Li, K. Huang and D. B. Qin, J. Solid State Chem., **2020**, *290*, 121561.
30. Y. Zhang, Q.F. Yang, X.P. Li, C.X. Miao, Q. Hou and S. Y. Ai, CrystEngComm., **2020**, *22*, 5690–5697.
31. X. J. Cao, X.L. Zhang, M.D. Zhang and J. S. Hu, Inorg. Chim. Acta., **2020**, *511*, 119836.
32. X. Y. Liang, Y.J. Jia, Z.Y. Zhan and M. Hu, Appl. Organomet. Chem., **2019**, *33*, e4988.
33. R. Rajak, M. Saraf, S.K. Verma, R. Kumar and S. M. Mobin, Inorg. Chem., **2019**, *58*, 16065–16074.
34. X. L. Zhang, J.S. Hu, B. Wang, Z.Q. Li, S.B. Xu, Y.N. Chen and X. M. Ma, J. Solid State Chem., **2019**, *269*, 459–464.
35. M. N. Peng, K. Huang, X.L. Li, D.F. Han, Q. Qiu, L.H. Jing and D. B. Qin, J. Solid State Chem., **2019**, *280*, 156–163.
